# Supplementary material for: Highly efficient coherent amplification of zero-field spin waves in YIG nanowaveguides
Source: Sci Adv. 2025 Sep 17;11(38):eadx2018. doi: 10.1126/sciadv.adx2018 (PMC12442858; doi:10.1126/sciadv.adx2018)
Supplement: Supplementary file 1 — Supplementary Text Figs. S1 and S2 References [file sciadv.adx2018_sm.pdf]

Science Advances
AAAS

Supplementary Materials for

Highly efficient coherent amplification of zero-field spin waves in YIG nanowaveguides

Kirill O. Nikolaev *et al.*

Corresponding author: Vladislav E. Demidov, demidov@uni-muenster.de

Sci. Adv. **11**, eadx2018 (2025)
DOI: 10.1126/sciadv.adx2018

This PDF file includes:

Supplementary Text
Figs. S1 and S2
References

Supplementary Text

Dispersion spectrum of spin waves at zero bias magnetic field

To characterize the dispersion spectrum, we perform phase-resolved BLS measurements at different frequencies of the microwave signal applied to the input antenna and determine the corresponding wavelength of spin waves and their wavenumber from Fourier analysis of the measured spatial dependences. The results of these measurements are shown in Fig. S1 by symbols. We additionally obtain the dispersion curve from the micromagnetic simulations (curve in Fig. S1) using the MuMax3 package (*43*) and the method described in detail in (*27*). In the simulations, we use the standard for YIG saturation magnetization, $4\pi M_s$ = 1750 G and the nominal YIG thickness of 80 nm. The width of the waveguide w is used as an adjustable parameter. The best agreement with the experimental data is achieved for w = 490 nm, which is very close to the nominal value of 500 nm.

Dependence of the ellipticity of the magnetization precession on the bias magnetic field

We additionally use micromagnetic simulations to calculate the ellipticity ε of the magnetization precession in a spin wave at different static magnetic fields applied parallel to the axis of the waveguide. We determine the smallest and largest values of the dynamic magnetization over the precession cycle, $m_{\min}$ and $m_{\max}$, and use the standard expression $\varepsilon = 1 - \frac{|m_{\min}|^2}{|m_{\max}|^2}$. Figure S2 shows the results of the calculations for a spin wave with a wavelength of 1.6 μm, which corresponds to a frequency of 1.5 GHz at zero bias magnetic field.

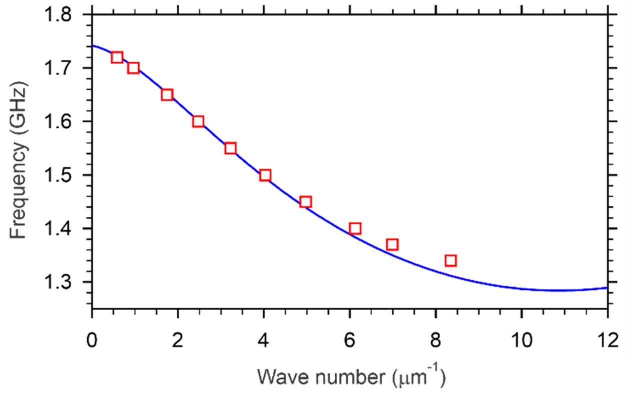

Fig. S1. Dispersion spectrum of spin waves at zero bias magnetic field. Symbols show the experimental data. Curve shows the result of micromagnetic simulations.

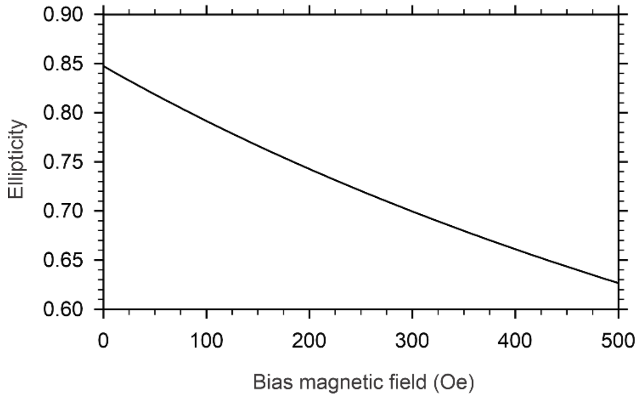

Fig. S2. Calculated field dependence of the ellipticity of the magnetization precession for a spin wave with a wavelength 1.6 μm.

REFERENCES AND NOTES

1. B. Flebus, D. Grundler, B. Rana, Y. Otani, I. Barsukov, A. Barman, G. Gubbiotti, P. Landeros, J. Akerman, U. Ebels, P. Pirro, V. E. Demidov, K. Schultheiss, G. Csaba, Q. Wang, F. Ciubotaru, D. E. Nikonov, P. Che, R. Hertel, T. Ono, D. Afanasiev, J. Mentink, T. Rasing, B. Hillebrands, S. V. Kusminskiy, W. Zhang, C. R. Du, A. Finco, T. van der Sar, Y. K. Luo, Y. Shiota, J. Sklenar, T. Yu, J. Rao, The 2024 magnonics roadmap. *J. Phys. Condens. Matter* **36**, 363501 (2024).

2. H. Yu, O. d'Allivy Kelly, V. Cros, R. Bernard, P. Bortolotti, A. Anane, F. Brandl, F. Heimbach, D. Grundler, Approaching soft x-ray wavelengths in nanomagnet-based microwave technology. *Nat. Commun.* **7**, 11255 (2016).

3. C. Liu, J. Chen, T. Liu, F. Heimbach, H. Yu, Y. Xiao, J. Hu, M. Liu, H. Chang, T. Stueckler, S. Tu, Y. Zhang, Y. Zhang, P. Gao, Z. Liao, D. Yu, K. Xia, N. Lei, W. Zhao, M. Wu, Long-distance propagation of short-wavelength spin waves. *Nat. Commun.* **9**, 738 (2018).

4. V. Sluka, T. Schneider, R. A. Gallardo, A. Kákay, M. Weigand, T. Warnatz, R. Mattheis, A. Roldán-Molina, P. Landeros, V. Tiberkevich, A. Slavin, G. Schütz, A. Erbe, A. Deac, J. Lindner, J. Raabe, J. Fassbender, S. Wintz, Emission and propagation of 1D and 2D spin waves with nanoscale wavelengths in anisotropic spin textures. *Nat. Nanotechnol.* **14**, 328–333 (2019).

5. K. Vogt, F. Y. Fradin, J. E. Pearson, T. Sebastian, S. D. Bader, B. Hillebrands, A. P. Hoffmann, H. Schultheiss, Realization of a spin-wave multiplexer. *Nat. Commun.* **5**, 3727 (2014).

6. M. Evelt, L. Soumah, A. B. Rinkevich, S. O. Demokritov, A. Anane, V. Cros, J. Ben Youssef, G. de Loubens, O. Klein, P. Bortolotti, V. E. Demidov, Emission of coherent propagating magnons by insulator-based spin-orbit-torque oscillators. *Phys. Rev. Appl.* **10**, 041002 (2018).

7. H. Qin, R. B. Holländer, L. Flajšman, F. Hermann, R. Dreyer, G. Woltersdorf, S. van Dijken, Nanoscale magnonic Fabry-Perot resonator for low-loss spin-wave manipulation. *Nat. Commun.* **12**, 2293 (2021).

8. T. Nozaki, Y. Shiota, S. Miwa, S. Murakami, F. Bonell, S. Ishibashi, H. Kubota, K. Yakushiji, T. Saruya, A. Fukushima, S. Yuasa, T. Shinjo, Y. Suzuki, Electric-field-induced ferromagnetic resonance excitation in an ultrathin ferromagnetic metal layer. *Nat. Phys.* **8**, 491–496 (2012).

9. R. Verba, M. Carpentieri, G. Finocchio, V. Tiberkevich, A. Slavin, Excitation of spin waves in an in-plane-magnetized ferromagnetic nanowire using voltage-controlled magnetic anisotropy. *Phys. Rev. Appl.* **7**, 064023 (2017).

10. S. Choudhury, A. K. Chaurasiya, A. K. Mondal, B. Rana, K. Miura, H. Takahashi, Y. Otani, A. Barman, Voltage controlled on-demand magnonic nanochannels. *Sci. Adv.* **6**, eaba5457 (2020).

11. G. Csaba, A. Papp, W. Porod, Perspectives of using spin waves for computing and signal processing. *Phys. Lett. A* **381**, 1471–1476 (2017).

12. A. Mahmoud, F. Ciubotaru, F. Vanderveken, A. V. Chumak, S. Hamdioui, C. Adelmann, S. Cotofana, Introduction to spin wave computing. *J. Appl. Phys.* **128**, 161101 (2020).

13. Q. Wang, G. Csaba, R. Verba, A. V. Chumak, P. Pirro, Nanoscale magnonic networks. *Phys. Rev. Appl.* **21**, 040503 (2024).

14. A. V. Sadovnikov, S. A. Odintsov, E. N. Beginin, S. E. Sheshukova, Yu. P. Sharaevskii, S. A. Nikitov, Toward nonlinear magnonics: Intensity-dependent spin-wave switching in insulating side-coupled magnetic stripes. *Phys. Rev. B* **96**, 144428 (2017).

15. H. Merbouche, B. Divinskiy, K. O. Nikolaev, C. Kaspar, W. H. P. Pernice, D. Gouéré, R. Lebrun, V. Cros, J. Ben Youssef, P. Bortolotti, A. Anane, S. O. Demokritov, V. E. Demidov, Giant nonlinear self-phase modulation of large-amplitude spin waves in microscopic YIG waveguides. *Sci. Rep.* **12**, 7246 (2022).

16. R. Dreyer, A. F. Schäffer, H. G. Bauer, N. Liebing, J. Berakdar, G. Woltersdorf, Imaging and phase-locking of non-linear spin waves. *Nat. Commun.* **13**, 4939 (2022).

17. Q. Wang, R. Verba, B. Heinz, M. Schneider, O. Wojewoda, K. Davídková, K. Levchenko, C. Dubs, N. J. Mauser, M. Urbánek, P. Pirro, A. V. Chumak, Deeply nonlinear excitation of self-normalized short spin waves. *Sci. Adv.* **9**, eadg4609 (2023).

18. K. O. Nikolaev, S. R. Lake, G. Schmidt, S. O. Demokritov, V. E. Demidov, Resonant generation of propagating second-harmonic spin waves in nano-waveguides. *Nat. Commun.* **15**, 1827 (2024).

19. S. Watt, M. Kostylev, Reservoir computing using a spin-wave delay-line active-ring resonator based on yttrium-iron-garnet film. *Phys. Rev. Appl.* **13**, 034057 (2020).

20. A. Papp, W. Porod, G. Csaba, Nanoscale neural network using non-linear spin-wave interference. *Nat. Commun.* **12**, 6244 (2021).

21. L. Körber, C. Heins, T. Hula, J.-V. Kim, S. Thlang, H. Schultheiss, J. Fassbender, K. Schultheiss, Pattern recognition in reciprocal space with a magnon-scattering reservoir. *Nat. Commun.* **14**, 3954 (2023).

22. A. Litvinenko, R. Khymyn, V. H. González, R. Ovcharov, A. A. Awad, V. Tyberkevych, A. Slavin, J. Åkerman, A spinwave Ising machine. *Commun. Phys.* **6**, 227 (2023).

23. H. Chang, P. Li, W. Zhang, T. Liu, A. Hoffmann, L. Deng, M. Wu, Nanometer-thick yttrium iron garnet films with extremely low damping. *IEEE Magn. Lett.* **5**, 6700104 (2014).

24. H. Yu, O. d’Allivy Kelly, V. Cros, R. Bernard, P. Bortolotti, A. Anane, F. Brandl, R. Huber, I. Stasinopoulos, D. Grundler, Magnetic thin-film insulator with ultra-low spin wave damping for coherent nanomagnonics. *Sci. Rep.* **4**, 6848 (2014).

25. C. Hauser, T. Richter, N. Homonnay, C. Eisenschmidt, M. Qaid, H. Deniz, D. Hesse, M. Sawicki, S. G. Ebbinghaus, G. Schmidt, Yttrium iron garnet thin films with very low damping obtained by recrystallization of amorphous material. *Sci. Rep.* **6**, 20827 (2016).

26. D. Breitbach, M. Schneider, B. Heinz, F. Kohl, J. Maskill, L. Scheuer, R. O. Serha, T. Brächer, B. Lägel, C. Dubs, V. S. Tiberkevich, A. N. Slavin, A. A. Serga, B. Hillebrands, A. V. Chumak,

P. Pirro, Stimulated amplification of propagating spin waves. *Phys. Rev. Lett.* **131**, 156701 (2023).

27. H. Merbouche, B. Divinski, D. Gouéré, R. Lebrun, A. El Kanj, V. Cros, P. Bortolotti, A. Anane, S. O. Demokritov, V. E. Demidov, True amplification of spin waves in magnonic nano-waveguides. *Nat. Commun.* **15**, 1560 (2024).

28. Q. Wang, R. Verba, K. Davídková, B. Heinz, S. Tian, Y. Rao, M. Guo, X. Guo, C. Dubs, P. Pirro, A. V. Chumak, All-magnonic repeater based on bistability. *Nat. Commun.* **15**, 7577 (2024).

29. A. G. Gurevich, G. A. Melkov, *Magnetization Oscillations and Waves* (CRC, New York, 1996).

30. A. V. Bagada, G. A. Melkov, A. A. Serga, A. N. Slavin, Parametric interaction of dipolar spin wave solitons with localized electromagnetic pumping. *Phys. Rev. Lett.* **79**, 2137–2140 (1997).

31. P. A. Kolodin, P. Kabos, C. E. Patton, B. A. Kalinikos, N. G. Kovshikov, M. P. Kostylev, Amplification of microwave magnetic envelope solitons in thin yttrium iron garnet films by parallel pumping. *Phys. Rev. Lett.* **80**, 1976–1979 (1998).

32. G. A. Melkov, A. A. Serga, A. N. Slavin, V. S. Tiberkevich, A. N. Oleinik, A. V. Bagada, Parametric interaction of magnetostatic waves with a nonstationary local pump. *J. Exp. Theor. Phys.* **89**, 1189–1199 (1999).

33. P. Chowdhury, P. Dhagat, A. Jander, Parametric amplification of spin waves using acoustic waves. *IEEE Trans. Mag.* **51**, 1300904 (2015).

34. T. Brächer, P. Pirro, T. Meyer, F. Heussner, B. Lägel, A. A. Serga, B. Hillebrands, Parallel parametric amplification of coherently excited propagating spin waves in a microscopic $Ni_{81}Fe_{19}$ waveguide. *Appl. Phys. Lett.* **104**, 202408 (2014).

35. B. Heinz, M. Mohseni, A. Lentfert, R. Verba, M. Schneider, B. Läge, K. Levchenko, T. Brächer, C. Dubs, A. V. Chumak, P. Pirro, Parametric generation of spin waves in nanoscaled magnonic conduits. *Phys. Rev. B* **105**, 144424 (2022).

36. K. Wagner, A. Kákay, K. Schultheiss, A. Henschke, T. Sebastian, H. Schultheiss, Magnetic domain walls as reconfigurable spin-wave nanochannels. *Nat. Nanotechnol.* **11**, 432–436 (2016).

37. A. Haldar, D. Kumar, A. O. Adeyeye, A reconfigurable waveguide for energy-efficient transmission and local manipulation of information in a nanomagnetic device. *Nat. Nanotechnol.* **11**, 437–443 (2016).

38. L. Flajšman, K. Wagner, M. Vaňatka, J. Gloss, V. Křižáková, M. Schmid, H. Schultheiss, M. Urbánek, Zero-field propagation of spin waves in waveguides prepared by focused ion beam direct writing. *Phys. Rev. B* **101**, 014436 (2020).

39. J. Klíma, O. Wojewoda, V. Roučka, T. Molnár, J. Holobrádek, M. Urbánek, Zero-field spin wave turns. *Appl. Phys. Lett.* **124**, 112404 (2024).

40. K. O. Nikolaev, S. R. Lake, G. Schmidt, S. O. Demokritov, V. E. Demidov, Zero-field spin waves in YIG nanowaveguides. *Nano Lett.* **23**, 8719–8724 (2023).

41. V. E. Demidov, S. O. Demokritov, Magnonic waveguides studied by microfocus Brillouin light scattering. *IEEE Trans. Mag.* **51**, 0800215 (2015).

42. G. A. Melkov, A. A. Serga, V. S. Tiberkevich, A. N. Oliynyk, A. N. Slavin, Wave front reversal of a dipolar spin wave pulse in a nonstationary three-wave parametric interaction. *Phys. Rev. Lett.* **84**, 3438–3441 (2000).

43. A. Vansteenkiste, J. Leliaert, M. Dvornik, M. Helsen, F. Garcia-Sanchez, B. Van Waeyenberge, The design and verification of MuMax3. *AIP Adv.* **4**, 107133 (2014).
